# Supplementary material for: MetaRibo-Seq measures translation in microbiomes
Source: Nat Commun. 2020 Jun 29;11:3268. doi: 10.1038/s41467-020-17081-z (PMC7324362; doi:10.1038/s41467-020-17081-z)
Supplement: Supplementary file 10 — Supplementary Data 7 [file 41467_2020_17081_MOESM10_ESM.zip › File2/Confidence_High_Taxonomy/259755_out.krona.html]

Javascript must be enabled to view this page.

members
magnitude
magnitudeUnassigned
count
unassigned
taxon
rank

259755\_out

217

217
2
superkingdom

217
1239
phylum

class
186801
217

order

SRS143466\_contig\_number\_206
1
217
186802

186803
155
3
family

SRS014235\_contig\_number\_contig-100\_2708.253046SRS142503\_contig\_number\_57298SRS143780\_contig\_number\_29608


SRS018427\_contig\_number\_30148
genus
1
22
189330

1262872
1

SRS024549\_contig\_number\_5737
species

9
88431
species

SRS013687\_contig\_number\_23172SRS015431\_contig\_number\_70343SRS018313\_contig\_number\_contig-100\_3085.77649SRS042966\_contig\_number\_contig-100\_5450.86124SRS1041130\_contig\_number\_contig-100\_180.50120SRS144714\_contig\_number\_29328SRS147977\_contig\_number\_contig-100\_726.92494SRS148159\_contig\_number\_58438SRS149879\_contig\_number\_contig-100\_364.159535

1263073
3

SRS018984\_contig\_number\_17104SRS049773\_contig\_number\_contig-100\_13.210054SRS1055067\_contig\_number\_12557
species

8
39486

SRS019161\_contig\_number\_contig-100\_2721.205287SRS054956\_contig\_number\_15400SRS064973\_contig\_number\_55SRS1041037\_contig\_number\_3037SRS1041091\_contig\_number\_contig-100\_867.120684SRS1041138\_contig\_number\_8868SRS1041142\_contig\_number\_9318SRS147919\_contig\_number\_19996
species

11
841
genus

1262945
3
species

SRS023715\_contig\_number\_contig-100\_6621.145067SRS046717\_contig\_number\_contig-100\_6319.68497SRS104636\_contig\_number\_contig-100\_14492.63256

5
166486

SRS063040\_contig\_number\_47149SRS076929\_contig\_number\_contig-100\_1279.149105SRS077454\_contig\_number\_19608SRS098061\_contig\_number\_162SRS143722\_contig\_number\_14330
species


SRS142542\_contig\_number\_25698
species
1
2293144

360807
1

SRS011084\_contig\_number\_38010
species

1262940
1
species

SRS017191\_contig\_number\_13672


SRS014534\_contig\_number\_contig-100\_7.14092SRS014948\_contig\_number\_16168SRS015217\_contig\_number\_30261SRS017821\_contig\_number\_contig-100\_1957.35400SRS019178\_contig\_number\_4811SRS022137\_contig\_number\_20864SRS042284\_contig\_number\_1894SRS043667\_contig\_number\_1600SRS046717\_contig\_number\_2965SRS050026\_contig\_number\_5223SRS051882\_contig\_number\_7621SRS054590\_contig\_number\_1010SRS055982\_contig\_number\_13518SRS065504\_contig\_number\_33796SRS098571\_contig\_number\_67355SRS142712\_contig\_number\_contig-100\_61.47202SRS144183\_contig\_number\_contig-100\_671.190463SRS146812\_contig\_number\_60667SRS147766\_contig\_number\_20386SRS893172\_contig\_number\_13770SRS893187\_contig\_number\_contig-100\_209.103984SRS893231\_contig\_number\_contig-100\_15.49623
species
39491
22

3
1898203

SRS043768\_contig\_number\_contig-100\_1019.186335SRS049995\_contig\_number\_contig-100\_897.209784SRS078176\_contig\_number\_contig-100\_736.138797
species

genus
33042
6

species

SRS020233\_contig\_number\_33490
1
2293088

458253
5
species

SRS043768\_contig\_number\_26695SRS053335\_contig\_number\_17559SRS077194\_contig\_number\_4426SRS1055076\_contig\_number\_contig-100\_150.89439SRS893378\_contig\_number\_23147

1
830
genus

species

SRS104693\_contig\_number\_13027
1
45851

26
1952029
species

SRS014235\_contig\_number\_6593SRS014412\_contig\_number\_18119SRS014979\_contig\_number\_13620SRS015694\_contig\_number\_13588SRS019910\_contig\_number\_contig-100\_8577.113796SRS044535\_contig\_number\_contig-100\_2802.144903SRS046502\_contig\_number\_contig-100\_27840.27841SRS049773\_contig\_number\_37924SRS050925\_contig\_number\_contig-100\_332.193133SRS052697\_contig\_number\_20217SRS058770\_contig\_number\_7727SRS062701\_contig\_number\_contig-100\_2003.69376SRS075021\_contig\_number\_1626SRS075341\_contig\_number\_contig-100\_134.109198SRS076976\_contig\_number\_contig-100\_7900.7901SRS104036\_contig\_number\_contig-100\_30196.30197SRS1041147\_contig\_number\_contig-100\_920.32370SRS140492\_contig\_number\_22628SRS142599\_contig\_number\_contig-100\_1360.185860SRS143417\_contig\_number\_contig-100\_15457.132496SRS143598\_contig\_number\_contig-100\_4968.133178SRS144506\_contig\_number\_53399SRS146813\_contig\_number\_27095SRS147022\_contig\_number\_contig-100\_681.79863SRS147614\_contig\_number\_contig-100\_2379.66665SRS148511\_contig\_number\_17738

1506553
10
genus


SRS012273\_contig\_number\_40826SRS014613\_contig\_number\_14954SRS015065\_contig\_number\_37395SRS019397\_contig\_number\_contig-100\_1996.74240SRS024435\_contig\_number\_44703SRS045004\_contig\_number\_contig-100\_18082.18082SRS045244\_contig\_number\_contig-100\_105.53079SRS050422\_contig\_number\_35063SRS056259\_contig\_number\_26617SRS143991\_contig\_number\_23189
species
1531
10

species

SRS014459\_contig\_number\_41846SRS078242\_contig\_number\_30722
2
2109691

species

SRS077086\_contig\_number\_6714
1
397290

48
572511

SRS148253\_contig\_number\_contig-100\_1270.97678
genus
1

26
1322
species

SRS011134\_contig\_number\_52161SRS014412\_contig\_number\_contig-100\_208.123460SRS015578\_contig\_number\_35783SRS015782\_contig\_number\_32370SRS015890\_contig\_number\_23153SRS017916\_contig\_number\_4502SRS019068\_contig\_number\_19260SRS020328\_contig\_number\_contig-100\_1384.185708SRS024388\_contig\_number\_4742SRS024663\_contig\_number\_5601SRS043841\_contig\_number\_11279SRS049959\_contig\_number\_45550SRS050752\_contig\_number\_6436SRS052027\_contig\_number\_20600SRS078419\_contig\_number\_35541SRS097889\_contig\_number\_41406SRS1041095\_contig\_number\_6148SRS1041137\_contig\_number\_11278SRS1055022\_contig\_number\_contig-100\_51.140794SRS144537\_contig\_number\_contig-100\_2742.253064SRS146764\_contig\_number\_33872SRS147377\_contig\_number\_9325SRS147614\_contig\_number\_10629SRS893288\_contig\_number\_contig-100\_62.36645SRS893295\_contig\_number\_contig-100\_241.73886SRS893383\_contig\_number\_36776

15
40520
species

SRS016267\_contig\_number\_839SRS021219\_contig\_number\_4406SRS024009\_contig\_number\_6092SRS045195\_contig\_number\_2604SRS048981\_contig\_number\_6793SRS058070\_contig\_number\_contig-100\_15.184700SRS062654\_contig\_number\_26818SRS074964\_contig\_number\_5714SRS098644\_contig\_number\_31390SRS1041112\_contig\_number\_6555SRS1041132\_contig\_number\_8191SRS104400\_contig\_number\_55960SRS143342\_contig\_number\_14430SRS148874\_contig\_number\_16268SRS893170\_contig\_number\_11001

33039
5
species

SRS014412\_contig\_number\_contig-100\_727.123979SRS014855\_contig\_number\_7810SRS077086\_contig\_number\_contig-100\_1420.72138SRS144135\_contig\_number\_34733SRS893259\_contig\_number\_5899

1
765821
species

SRS1054691\_contig\_number\_1224

186806
3
family

1730
3
genus

3
142586
species

SRS019496\_contig\_number\_14708SRS143148\_contig\_number\_23912SRS148784\_contig\_number\_14590

family
541000
7

1
1898205
species

SRS011302\_contig\_number\_19517

1263
6
genus

1
2293194
species

SRS045826\_contig\_number\_15774

46228
1

SRS144537\_contig\_number\_contig-100\_1329.251651
species

2293184
1
species

SRS1041039\_contig\_number\_11537


SRS063518\_contig\_number\_37940SRS1041146\_contig\_number\_contig-100\_21.18500SRS1054928\_contig\_number\_6317
species
165186
3

51
31979
family

1485
51
1

SRS013965\_contig\_number\_contig-100\_1478.167722
genus

2293010
1

SRS045713\_contig\_number\_17008
species


SRS013476\_contig\_number\_contig-100\_301.132913SRS013800\_contig\_number\_16457SRS014287\_contig\_number\_contig-100\_2631.122710SRS015486\_contig\_number\_1115SRS018351\_contig\_number\_16710SRS022524\_contig\_number\_15900SRS024132\_contig\_number\_18282SRS043001\_contig\_number\_8104SRS053573\_contig\_number\_contig-100\_803.803SRS056273\_contig\_number\_32278SRS062701\_contig\_number\_contig-100\_2004.69377SRS104084\_contig\_number\_13142SRS104327\_contig\_number\_contig-100\_4783.4784SRS140513\_contig\_number\_15559SRS143598\_contig\_number\_15311SRS146764\_contig\_number\_contig-100\_2906.199272SRS148253\_contig\_number\_contig-100\_2522.98930SRS148970\_contig\_number\_contig-100\_12863.12864
species
18
665940


SRS013521\_contig\_number\_14673SRS014736\_contig\_number\_9336SRS019693\_contig\_number\_1790SRS056519\_contig\_number\_3194SRS098717\_contig\_number\_18548SRS146888\_contig\_number\_7703
species
1262842
6

8
2292993
species

SRS042628\_contig\_number\_20404SRS048164\_contig\_number\_31700SRS053214\_contig\_number\_13025SRS077127\_contig\_number\_17814SRS104311\_contig\_number\_contig-100\_2239.252342SRS104485\_contig\_number\_4466SRS149181\_contig\_number\_26604SRS893300\_contig\_number\_5993

species

SRS064276\_contig\_number\_42528
1
1262792


SRS012849\_contig\_number\_28998SRS015663\_contig\_number\_30070SRS016095\_contig\_number\_29508SRS053356\_contig\_number\_contig-100\_1752.269267SRS055017\_contig\_number\_36850SRS142923\_contig\_number\_23222SRS147346\_contig\_number\_33140
species
2305242
7

species

SRS021484\_contig\_number\_31946SRS104197\_contig\_number\_contig-100\_660.660
2
1262824

2
2293002

SRS014287\_contig\_number\_18757SRS014923\_contig\_number\_45307
species

59620
2

SRS105153\_contig\_number\_27494SRS142781\_contig\_number\_12046
species

species

SRS013951\_contig\_number\_19571
1262845
1

species

SRS015264\_contig\_number\_17781SRS077552\_contig\_number\_17000
2
2293051
